# Proceedings Central Texas Medical Society

**Published:** 1889-05

**Authors:** W. O. Wilkes

**Affiliations:** Secretary


### Society Notes.

### PROCEEDINGS CENTRAL TEXAS MEDICAL ASSOCIATION.

*Editor Daniel's Texas Medical Journal*:

The Central Texas Medical Association held by far the most interesting meeting in its history, in this city on Tuesday, April 9, 1889. The attendance was very large; at least fifty out-of-town physicians being present. Seventeen additions were made to the membership, and one application was rejected by the Board of Censors. The Association is auxilliary to the State Medical Association, and the requirements for membership are just the same as in the State Association. Papers were read as follows:

"Malarial Hæmaturia," by Dr. W. A. Howard.

"A Strange Case of Gangrene," by Dr. R. W. Park.

"Antiseptic in Obstetrics," by Dr. J. H. Sears, followed by Dr. A. M. Curtis, with a paper on the same subject. These two gentlemen were at opposite extremes on this subject, and the discussion which followed the reading of the two papers was very interesting.

"Malignant Abdominal Tumor in a Boy," by Dr. W. L. Barker.

"Treatment of Rupture of the Uterus," by Prof. W. H. Wathen, of Louisville, Ky.

"Pernicious Vomiting of Pregnancy," by Dr. S. E. Shelton.

The discussions which followed the reading of these papers were long, interesting and instructive, and were enjoyed by all present.

The next meeting will take place in Waco, Tuesday, July 9, 1889, for which session the following subjects and authors have been selected :

"Puerperal Eclampsia," Dr. J. C. Shaw. "Placenta Prævia," Dr. W. H. Wilkes. "The Antipyretics," Dr. C. F. Paine. "Fractures of the Femur," Dr. A. H. Snead.

Dr. J. W. Cock will continue the subject of "Tobacco Chewing as a Preventive for Consumption."

Volunteer papers are desired, and it is requested that their titles be sent in as early as possible, so that they may be printed on the notices to be sent out shortly before the meeting.

Very respectfully,

W. O. WILKES, M. D.,

Secretary.

---

THE Ohio State Medical Society will meet in Youngstown, Ohio, May 22nd, 23rd and 24th inst. Dr. G. A. Collamore, secretary, Toledo, Ohio.

